# Fibrinogen facilitates atherosclerotic formation in Sprague-Dawley rats: A rodent model of atherosclerosis

**DOI:** 10.3892/etm.2013.913

**Published:** 2013-01-21

**Authors:** BIRONG ZHOU, YING PAN, QIANQIAN YU, ZHIMIN ZHAI

**Affiliations:** 1 Department of Cardiology, The First Affiliated Hospital of Anhui Medical University, Hefei, Anhui 230022;; 2 Department of Hematology, The Second Affiliated Hospital of Anhui Medical University, Hefei, Anhui 230601, P.R. China

**Keywords:** Sprague-Dawley rat, fibrinogen, atherosclerosis

## Abstract

Fibrinogen (Fg) contributes to thrombosis and hemostasis and plays a role in inflammation. Fg is also known to play a significant role in atherosclerosis (AS). P-selectin has been associated with AS. The present study aimed to identify the role of Fg in AS and to examine the possible mechanisms behind the effects of fibrinogen on AS using Sprague-Dawley (SD) rats as a model system. Diet-induced atherosclerotic SD rats were adopted as the experimental models. Fg was transfused into these rats and the degree of atherosclerotic lesion development was compared with that of control rats. Blood was obtained from the common abdominal aorta and then the biochemical characteristics were measured and ELISA assays performed. The aortas were then carefully separated, removed and placed in 10% (w/v) neutral formalin for use at a later stage. The root of the aorta was cut and samples were washed, dehydrated, cleared, dipped in wax, embedded, sliced, coated, grilled and stained with HE. Pathological HE-stained sections were examined by light microscopic analysis and immunohistochemistry was performed for Fg and P-selectin on representative tissue sections. The Fg-transfused, high-fat diet-fed group developed atherosclerotic lesions more readily compared with the control group. Immunohistochemical analysis revealed that Fg expression was higher in the endarterium of the Fg-transfused, high-fat diet-fed rats. P-selectin expression was also found to be correlated with Fg expression. Fg actively promotes atherosclerotic lesion development; one possible mechanism behind this is the ability of Fg to enhance P-selectin expression, which is also able to facilitate the development of atherosclerotic lesions.

## Introduction

It is well known that atherosclerosis (AS), characterized by an infiltration of leukocytes into the lesion foci, is one of the most widespread threats to human health and survival (1). The formation and development of AS lesions is a chronic and progressive process that requires a long time. AS is characterized by the accumulation of lipids and other elements in the coronary artery (2–5). However, the precise etiopathogenesis of the disease is currently unknown. The mechanisms behind the development of AS may include dyslipidemia and inflammation among other factors.

Fibrinogen (Fg) plays a significant role in homeostasis and thrombosis and is able to promote the formation of atherosclerotic plaques through various mechanisms. Fg promotes cell migration and adhesion. In addition, large amounts of fibrin, a metabolite of Fg, are located in atherosclerotic plaques and also promote the proliferation and migration of cells. Fibrin is also able to bind to fibronectin. Finally, fibrin in the inner layer is able to attract leukocytes and promote lipid accumulation in atherosclerotic plaques (6). Platelets and P-selectin also play significant roles in hemostasis and thrombosis. Evidence has shown that P-selectin is able to promote the development of atherosclerotic lesions (1,3,7). A study by Yang *et al*(7) reported for the first time that Fg may restore the surface expression of P-selectin in Fg-deficient (Fg-/-) mice. We thus hypothesized that Fg may regulate or control the expression of P-selectin during the formation and/or development of AS and thus facilitate atherosclerotic lesion development and promote plaque formation. In the present study, AS was successfully induced in Sprague-Dawley (SD) rats prior to Fg being trans-fused into them. Fg was shown to promote the development of atherosclerotic lesions and plaques, as rats that did not receive an Fg transfusion developed atherosclerotic lesions that were relatively small in comparison.

## Materials and methods

### Animal breeding and experimental protocol

SD rats (SPF, 200±10 g) were purchased from the Experimental Animal Center of Anhui, China. The rats were maintained in controlled temperature (21–23°C), light (12-h light, 12-h dark) and humidity (55±5%) conditions with access to food and water *ad libitum*. Subsequent to a 3-day adaptation period, they were randomly divided into 4 groups. Groups Z and ZF were fed a normal chow diet, while groups H and HF were fed a high-fat diet for ∼15 weeks. The high-fat diet contained 83.3% normal chow, 8% lard, 3% cholesterol, 5% plantation white sugar, 0.2% propylthiouracil and 0.5% chleolate. At the beginning of the experiment, vitamin D_2_ (3×10^5^ U/kg) was injected into the rats from group H and HF. In the 7th and 8th weeks, human Fg was injected intravenously at a dose of 2.0 mg per rat into the rats from groups ZF and HF. At the end of the 15th week, all animals were fasted for ≥8 h prior to being anesthetized with 10% chloral hydrate at a dose of 0.3 ml/100 g. Blood was obtained from the common abdominal aorta of the rats and biochemical characteristics were then measured and enzyme-linked immunosorbent assays (ELISAs) performed. The aortas were carefully separated, removed, cut open, observed and placed in 10% (w/v) neutral formalin for later use. All the animal experiments were conducted with approval from the Internal Animal Care and Use Committee of Anhui Medical University and in compliance with the Guide for the Care and Use of Laboratory Animals.

### Reagents

Unconjugated anti-rat P-selectin antibodies were purchased from Boiss (Beijing, China). Unconjugated anti-rat fibrinogen antibodies were purchased from Santa Cruz Biotechnology, Inc. (Santa Cruz, CA, USA). SP-9000/9001/9002 Histostain TM-Plus kits were purchased from Zymed (San Diego, CA, USA). All other chemicals used were commercially available and pure grade.

### Biochemical analyses

The blood fat concentrations were determined enzymatically using automatic biochemical analyzers.

### Morphological measurements (HE staining)

The aortas were stored in 10% (w/v) neutral formalin for at least one day as described. The roots of the aortas were cut off and then the samples were washed, dehydrated, cleared, dipped in wax, embedded, sliced, coated, grilled and stained with HE. Finally, the thickness of the intima and media were examined and photographed with an image operation system under a 1/10-mm eye lens and with 40×10 amplification.

### Immunohistochemistry

The aorta slices were dewaxed and washed with distilled water and then incubated in 3% hydrogen peroxide for 10 min to block the endogenous peroxidase. Subsequent to being washed with PBS, the aorta slices were incubated in 10% normal goat serum for 30 min to block unspecific binding. Mouse monoclonal primary antibodies against P-selectin or Fg were added and incubated overnight at 4°C. The samples were placed at 37°C for 30 min to return them to a normal temperature and then were incubated with a secondary antibody for 20 min at 37°C, followed by being washed 3 times with PBS for 3 min each time. Horseradish peroxidase (HRP; 100 *μ*l) was added to the slides which were then incubated at 37°C for 20 min. Following coloration with diaminobenzidine (DAB), the slides were processed with hematoxylin light staining for 2 min, followed by bluing, dehydration, clearing and mounting. Finally, the morphological changes in the vessel walls were observed and images were captured.

### Detection of plasma Fg by ELISA

Plasma Fg was measured using an Fg ELISA kit (R&D Systems, Minneapolis, MN, USA). The blood samples were collected into EDTA-coated cuvettes and centrifuged at 1,000 x g for 10 min to remove the cells and collect the plasma. All reagents were prepared prior to starting the assay procedure. As recommended by the manufacturer, all standards and samples were added in duplicate to the microELISA strip plate. A total of 50 *μ*l of each standard and 10 *μ*l of the testing samples diluted in 40 *μ*l of the sample dilution were used. A blank well with nothing added was also included. Next, 100 *μ*l of the HRP-conjugate reagent was added to each well and the plates were covered with an adhesive strip and incubated for 60 min at 37°C. Each well was aspirated and washed five times by filling the well with a wash solution (400 *μ*l) using a squirt bottle, manifold dispenser or autowasher. The liquid was removed completely at each step to ensure a good performance. Subsequent to the last wash, any remaining wash solution was removed by aspirating or decanting. The plate was then inverted and blotted against clean paper towels. Next, 50 *μ*l chromogen solution A and 50 *μ*l chromogen solution B were added to each well and the plate was gently mixed and incubated for 15 min at 37°C in the dark. After this, 50 *μ*l stop solution was added to each well. The color in the wells was observed to change from blue to yellow. If the color in the well was green or the color change did not appear uniform, the plates were gently tapped to ensure thorough mixing. The optical density (OD) was read at 450 nm using a microtiter plate reader within 15 min of the addition.

### Statistical analysis

All statistical analyses were performed using SPSS version 13.0 for Windows (SPSS, Inc., Chicago, IL, USA). All data are expressed as mean ± standard deviation (SD). Comparisons between the groups were carried out using a one-way analysis of variance (ANOVA) and the Student-Newman-Keuls (SNK) method. A value of P<0.05 was considered to indicate a statistically significant difference.

## Results

### Changes in the blood fat levels in rats from the various experimental groups

**Figure 1.**
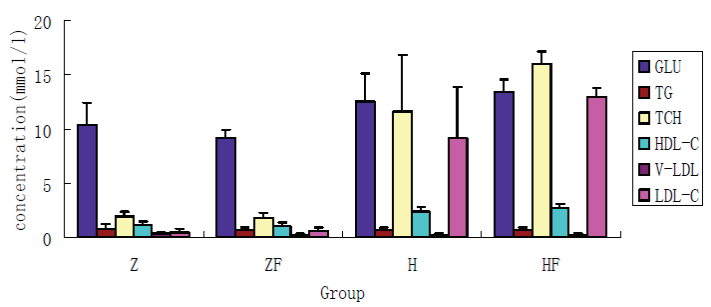
Changes in the blood fat and glucose levels in the various experimental groups. The serum TCH and LDL-C levels in the group Z and ZF were significantly lower than those of the group H and HF, there was a statistical significance (P<0.05). There was no statistically significant difference in the serum levels of TCH and LDL-C between groups H and HF or groups Z and ZF. GLU, glucose; TG, triglyceride; TCH, total cholesterol; HDL-C, high-density lipoprotein cholesterol; V-LDL, very low density lipoprotein; LDL-C, low-density lipoprotein cholesterol.

The induction of hypercholesterolemia was accompanied by an increase in the serum total cholesterol (TCH) and low-density lipoprotein cholesterol (LDL-C) levels. The serum lipoprotein analysis showed a dominant LDL-C fraction. The changes in the cholesterol and glucose levels of the rats following the various treatments for each group are shown in Fig. 1. As shown in Fig. 1, the serum TCH and LDL-C levels in the group Z and ZF were significantly lower than those of the group H and HF (P<0.05), there was statistical significance (P<0.05). However, there was no statistically significant difference in the serum levels of TCH and LDL-C between groups H and HF or groups Z and ZF.

### Plasma Fg levels

The plasma fibrinogen level was detected by ELISA in the four groups, there was significant differences in group H or group HF compared with group Z, there was a significant difference between group HF and group ZF, there was no difference between groups ZF and Z or groups HF and H (Table I).

### Light microscopic analysis of the pathological HE-stained sections

**Figure 2.**
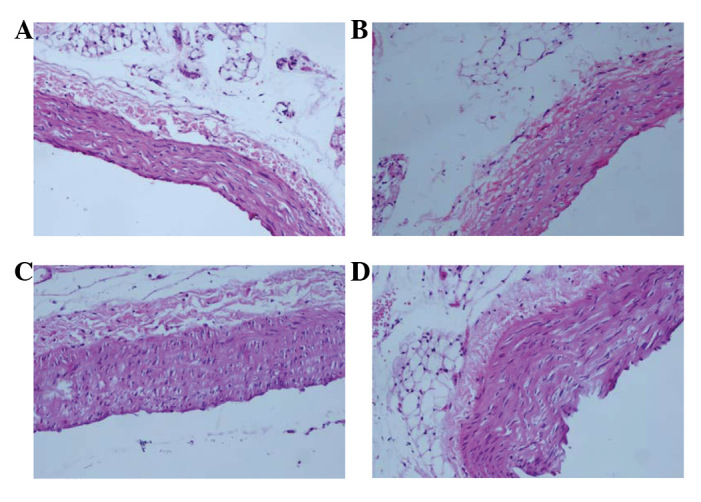
Light microscopic analysis of the pathological HE-stained aorta sections of rats from groups Z, ZF, H and HF (magnification, ×400). (A) In the control group (group Z), the vessel walls are thin and smooth with an even thickness. (B) In group ZF, the vessel walls are rough, but no foam cells are present. (C) In group H, numerous foam cells and atheronecrotic substances are present in the intima. (D) In group HF, more foam cells, atheronecrotic substances and calcification are present in the intima compared with in group H.

In the control group, group Z, the vessel walls were round and even in thickness. The inner and outer elastic plates were clear and complete and the endotheliocyte core was stained blue and evenly arranged. Also, no smooth muscle cells were observed underneath the endoderm (Fig. 2A). The vessel walls in group ZF were not as smooth as in group Z, but no foam cells were observed (Fig. 2B). The vessels in group H were rougher and thicker than those in groups Z and ZF and numerous foam and inflammatory cells were detected (Fig. 2C). The vessel walls in group HF were rough, the intima exhibited signs of hyperplasia and the thickness was uneven compared with group H. Numerous foam cells and atheronecrotic substances were observed under the fiber caps and cholesterol crystals and a few inflammatory cells were also observed there (Fig. 2D).

### Immunohistochemical staining of Fg and P-selectin

**Figure 3.**
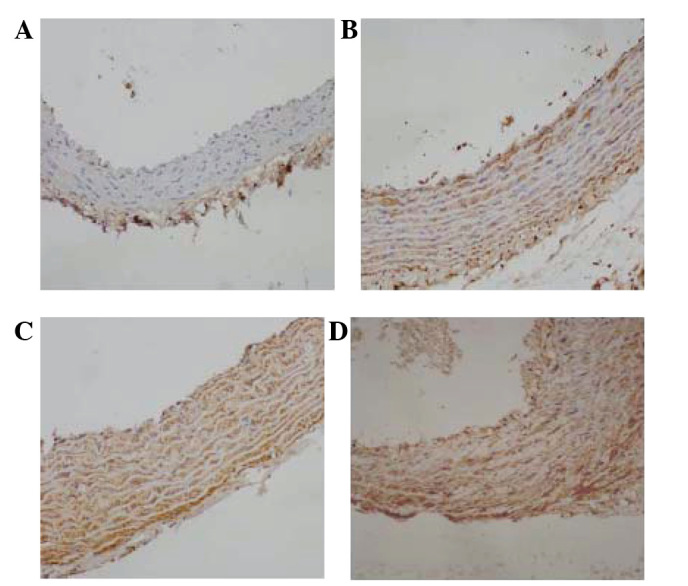
Fg expression in the various experimental groups (immunohistochemistry, ×200). (A) In the control group (group Z), little brown staining is present in the vessel walls. (B) In group ZF, limited brown staining is present in the endoderm of the vessel walls. (C) In group H, lots of brown staining is present in the intima. (D) In group HF, more widespread and dense brown staining is present in the vessel walls. Fg, fibrinogen.

**Figure 4.**
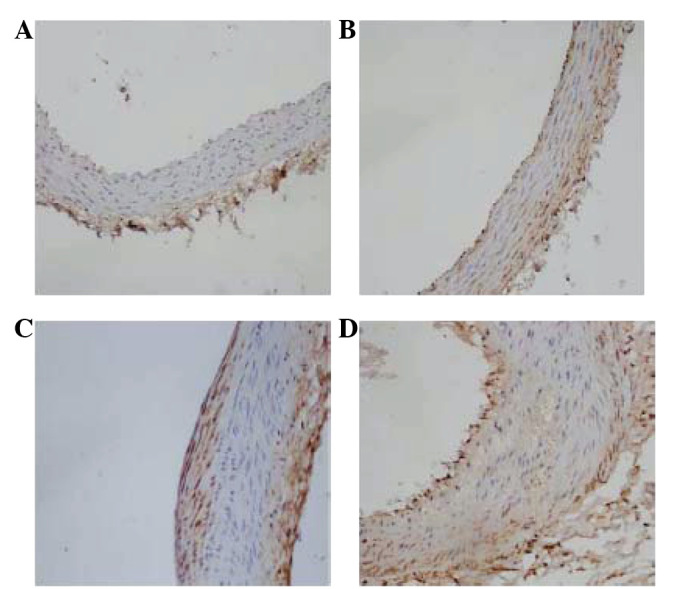
P-selectin expression in the various experimental groups (immunohistochemistry, ×200). (A) In the control group (group Z), little brown staining is present in the vessel walls; (B) In group ZF, limited brown staining is present in the endoderm of the vessel walls. (C) In group H, lots of brown staining is present in the intima. (D) In group HF, more widespread and dense brown staining is present in the vessel walls.

Immunohistochemistry was performed for Fg or P-selectin on representative tissue sections. Positive Fg or P-selectin staining was observed as a brown stain. In group Z, little brown staining was observed in the vessel walls, particularly in the endarterium, while a small amount of brown staining was observed underneath the endoderm in the rats of the ZF group (Figs. 3A and B and 4A and B). In group H, lots of positive staining was detected in the vessel walls (Figs. 3C and 4C). In group HF, Fg or P-selectin immunostaining resulted in a more widespread and dense smear, including brown staining in the nucleus (Figs. 3D and 4D).

## Discussion

Currently, cardiovascular and cerebrovascular disease, of which AS is a component, are two major causes of disability and mortality (2,3). The investigation into the etiopathogenesis and pathogenesis of AS and the development of effective measures to delay and reverse the progression of AS, thereby reducing mortality from cardiovascular and cerebrovascular diseases, has become an important field of study (8–10). However, AS has a complex, multifactorial pathophysiology and a number of risk factors work together to shape atherosclerotic plaques. The initial stage of AS is characterized by the infiltration and adherence of monocytes to the surface of the injured endothelium, for which adhesion molecules maybe indispensable.

Fg, which is produced by hepatocytes, is composed of three homologous polypeptide chains; α, β and γ. As the soluble precursor of fibrin, Fg is a 340-kDa hexamer (11) that has a central E domain, two peripheral D domains and three stranded coiled coils. The D domain contains a globular carboxyl end made up of β- and γ-chains and the E domain contains an α-terminus with 6 polypeptide chains. The E and D domains are linked by three stranded coiled coils (12,13). Fg is a key molecule in hemostasis and thrombosis and also plays a role in pathophysiological processes, including infection and wound healing. Fg also plays a significant role in the formation and progression of atherosclerotic plaques (12,14–18). It is becoming increasingly clear that fibrinogen is an inflammation marker for cardiovascular disease (6) that is expressed at the site of plaque ruptures (16).

It is also evident that Fg plays a significant role in AS. Certain possible mechanisms by which Fg affects AS have been identified (17,19). Firstly, Fg-fibrin composition is able to promote the formation of atherosclerotic plaques in AS by enhancing the deposition of lipids into the vessel walls, thereby attracting macrophagocytes which swallow the lipid material (12) and form foam cells. Secondly, Fg plays a key role in thrombosis, which causes plaque instability in AS. Thirdly, Fg is able to contribute to atherogenesis through interactions with the endothelial cells, smooth muscle cells and macrophages, while also playing a role in the transfer of cholesterol to the mononuclear cells and macrophages (19,20). Fg and fibrin are able to stimulate chemokine secretion and facilitate neutrophil-endothelial cell interactions. Numerous clinical experiments have demonstrated that Fg plays a critical role in the formation of plaques. Lepedda *et al*(16) reported that patients with unstable plaques had a higher level of plasma Fg than patients with stable plaques. This means that Fg may play a more significant role in the formation of unstable plaques than in stable plaques. A previous study demonstrated that an infusion of activated platelets caused the release of Weibel-Palade bodies leading to P-selectin-mediated leukocyte rolling. This suggested that P-selectin is crucial in the processes of inflammation and AS (15–17). Earlier studies (21,22,16,18) have clearly demonstrated that Fg enhances intracellular platelet P-selectin levels and affects P-selectin expression on the surface of mouse and human platelets. This may partially explain the role of Fg in inflammation, hemostasis and AS. P-selectin is a member of the selectin family of cell adhesion receptors and is also known as CD62P, GMP-140 or PADGEM (platelet activation-dependent granule external membrane) (23). P-selectin is localized to Weibel-Palade bodies in the endothelial cells or to α-granules in platelets (24). Platelet P-selectin is involved in multiple physiological processes, including platelet aggregation and platelet-leukocyte and platelet-endothelial cell interactions. Clinically, P-selectin is widely accepted as a marker of platelet activation, with the elevated levels of plasma P-selectin in thrombotic disorders resulting mainly from the shedding of P-selectin from the surface of the platelets. Studies have confirmed that P-selectin gene-deficient mice have a lower incidence of AS (25,26). Graff *et al*(27) discovered that P-selectin levels are highly associated with the release of platelet-derived growth factor (PDGF), which is one of the growth factors secreted by endothelial cells (ECs). The authors also confirmed that P-selectin may participate in the early stages of AS. However, there is as of yet little insight into the mechanisms that regulate platelet P-selectin expression. In the present study, Fg transfusion in the experimental ZF group led to a higher level of P-selectin expression compared with that observed in group Z, as determined by immunohistochemistry. Similar results were also obtained when groups H and HF were compared.

P-selectin may contribute to the formation of AS in several ways. P-selectin expression is associated with the adhesion of mononuclear cells to vessel walls and the subsequent formation of fatty streaks. P-selectin-mediated macrophage and T cell accumulation in the endarterium and platelet activation are also involved in the complications associated with AS. Variations in the sheer stress increase platelet P-selectin levels. The deposition of platelets into the extracellular matrix provides a leading adhesion site for the accumulation of molecules. The present study observed that it is easier to form plaques in certain crotch of grave vessels, this may be associated with the activation and deposition of platelets that were caused by the changes in hemodynamics and the high P-selectin expression (28,29).

In the present study, the expression of Fg and P-selectin in an atherosclerotic SD rat model system was examined. Differences in the Fg levels may account for the differences observed in the P-selectin levels, although the possibility of other effects attributed to Fg cannot be excluded. Fg was demonstrated as able to promote the development of AS lesions, while P-selectin levels, which also play a role in lesion development, were identified as positively correlated with Fg levels. Thus, we propose that affecting P-selectin expression levels may be one mechanism by which Fg participates in AS. These two factors may have a synergistic effect on the development of lesions and AS. It is therefore worthwhile to further investigate the correlation between Fg and platelet P-selectin expression in AS. In addition, variations in Fg concentration may significantly affect intracellular and cell surface platelet P-selectin expression.

In summary, Fg and P-selectin are crucial for the growth of atherosclerotic lesions. Thus, agents that inhibit Fg and/or P-selectin or that prevent platelet activation may become effective tools in reducing the development of atherosclerotic lesions. Moreover, further demonstrations of whether Fg actually affects P-selectin levels in AS, as well as a clarification of the mechanism behind such an action, require additional investigation. Further studies should also be conducted into whether other factors affect Fg and P-selectin expression or function.

## Figures and Tables

**Table I.** Plasma Fg (g/l) levels in rats from the various experimental groups (mean ± SD).

Group	Fg
Z	2.25±0.25
ZF	3.09±0.20
H	3.72±0.23^a^
HF	4.21±0.35^a,b^

a Significant difference from group Z (P<0.05).

b Significant difference from group ZF (P<0.05). Fg, fibrinogen.
