# [Expression of Concern] Fibrinogen facilitates atherosclerotic formation in Sprague-Dawley rats: A rodent model of atherosclerosis

**DOI:** 10.3892/etm.2025.12988

**Published:** 2025-10-01

**Authors:** Birong Zhou, Ying Pan, Qianqian Yu, Zhimin Zhai

Exp Ther Med 5:730–734, 2013; DOI: 10.3892/etm.2013.913

Following the publication of this paper, it was drawn to the Editor’s attention by a concerned reader that, for the immunohistochemistry experiments shown in Figs. 3 and 4, the panels showing the control group (group Z) experiments (Fig. 3A and Fig. 4A) featured the same data, where the results from apparently different experiments were intended to have been shown (Figs. 3 and 4 were reported to show the results of fibrinogen and P-selectin expression, respectively). The authors were contacted by the Editorial Office to either offer an explanation for this apparent anomaly in the presentation of the data in this paper, or to confirm that the data in these figures had been assembled correctly; however, up to this time, no response from them has been forthcoming. Owing to the fact that the Editorial Office has been made aware of potential issues surrounding the scientific integrity of this paper, we are issuing an Expression of Concern to notify readers of this potential problem while the Editorial Office continues to investigate this matter further.

